# Dynamic Reconfiguration of a RGBD Sensor Based on QoS and QoC Requirements in Distributed Systems

**DOI:** 10.3390/s150818080

**Published:** 2015-07-24

**Authors:** Eduardo Munera, Jose-Luis Poza-Lujan, Juan-Luis Posadas-Yagüe, José-Enrique Simó-Ten, Juan Fco. Blanes Noguera

**Affiliations:** University Institute of Control Systems and Industrial Computing (ai2), Polytechnic University of Valencia (UPV), Camino de Vera, Valencia 46022, Spain; E-Mails: emunera@ai2.upv.es (E.M.); jposadas@ai2.upv.es (J.-L.P.-Y.); jsimo@ai2.upv.es (J.-E.S.-T.); pblanes@ai2.upv.es (J.F.B.N.)

**Keywords:** RGBD sensor, system reconfiguration, quality of service (QoS), quality of context (QoC)

## Abstract

The inclusion of embedded sensors into a networked system provides useful information for many applications. A Distributed Control System (DCS) is one of the clearest examples where processing and communications are constrained by the client’s requirements and the capacity of the system. An embedded sensor with advanced processing and communications capabilities supplies high level information, abstracting from the data acquisition process and objects recognition mechanisms. The implementation of an embedded sensor/actuator as a Smart Resource permits clients to access sensor information through distributed network services. Smart resources can offer sensor services as well as computing, communications and peripheral access by implementing a self-aware based adaptation mechanism which adapts the execution profile to the context. On the other hand, information integrity must be ensured when computing processes are dynamically adapted. Therefore, the processing must be adapted to perform tasks in a certain lapse of time but always ensuring a minimum process quality. In the same way, communications must try to reduce the data traffic without excluding relevant information. The main objective of the paper is to present a dynamic configuration mechanism to adapt the sensor processing and communication to the client’s requirements in the DCS. This paper describes an implementation of a smart resource based on a Red, Green, Blue, and Depth (RGBD) sensor in order to test the dynamic configuration mechanism presented.

## 1. Introduction

In a Distributed Control System (DCS) [1], visual sensors usually offer the information in a raw data format. It means that Red, Green, and Blue (RGB) frames must be sent to every client, with the corresponding bandwidth consumption. Additionally, every frame must be processed by each client based on their own requirements, for example, to recognize a specific form or to detect a particular colour.

In this context, in the case that different clients need to obtain identical outcomes, as well as when they need to recognize exactly the same form with the same colour, they have to do the same processing tasks, which implies unnecessary and redundant processing and also unnecessary bandwidth consumption.

Nowadays, in order to reduce the processing load on the client side, networked visual sensors are evolving to provide more processed information by moving part of the processing from clients to the visual sensors. Consequently, bandwidth consumption can be reduced by sending processed information instead of raw data from the sensor. For example, a visual sensor provides only a message with the colour of a detected form instead of the full RGB frame. This type of visual sensors is included in the smart device paradigm [2] which defines a smart device as a sensor and/or an actuator with capacity of processing.

Using this model, in opposite to raw data clients and visual sensors need to increase the complexity of communications in order to configure the details of data processing, such as the colour to be detected by the visual sensor process, and to access visual sensors processed data. Consequently, clients will need a mechanism to configure the visual sensors depending on their requirements and, in the same way, the use of interfaces to access processed data will be required.

The introduction of new technologies is increasing the development with this type of visual sensors in the last years. For example, the Microsoft Kinect [3] or Asus Xtion with similar properties [4], provide RGB and depth information of frames. These visual sensors are known as RGBD sensors [5].

Possible applications of these RGBD sensors range from industrial applications [6] to consumer oriented products, which can be easily accessible through web-based services from a personal computer, smartphone and wearable tools [7], mobile robot platforms [8], unmanned aerial vehicles [9], perception systems [10], and people management [11] can also take advantage of distributed RGBD sensors to obtain processed information to satisfy the environment knowledge requirements of the system.

In certain applications, RGBD sensors can receive numerous petitions from different clients by requesting different type of information. For example, in robot navigation under uncertain environment conditions, where the context is changing dynamically, the information requested to detect people to avoid them or to recognize doors to generate a map could be very different in comparison. The processing load of the RGBD sensor depends on the number of client requests and their type. Anyway, the RGBD sensor should ensure some specific service requirements that depend on the internal constraints, as for example Central Processing Unit (CPU) load. These service requirements can be specified with the quality of context (QoC) [12] and quality of service (QoS) [13] parameters. A client requests a specific QoS, for example the minimal resolution for the images, and the RGBD sensor provides this QoS according to its QoC, for example, by adjusting the image resolution in order to not to exceed a specific CPU load. Therefore, the smart resource should have an internal mechanism that allows adapt the processing to the constraints according to QoS and QoC.

According to this, the main objective of the paper is to present a dynamic configuration mechanism to adapt the visual sensor processing and communication to the client requirements in the DCS. To achieve this objective, the following goals are established:

- Using a communications interface that provides an adequate level of abstraction to access to smart devices. The aim is that clients can access transparently to smart devices by means of resources provided by them. This introduces the proposed concept called smart resource, where clients can access resources regardless of the devices that produce the information and where they are placed.
- To structure the processing of a smart device by means of isolated processes called plugins. Plugins offer basic functions that can be composed between them to perform more complex functions depending on the processing required by the clients.
- To propose an internal adaptation mechanism based on plugins to configure smart devices according to the QoS and QoC.
- To characterize a RGBD sensor into a smart resource, based on the publish/subscribe paradigm [14], and to test the proposed internal adaptation mechanism by implementing a case of study.

The paper is organized as follows: in Section 2 some related work is introduced. The current framework is presented in Section 3. Context adaptation mechanisms are detailed in Section 4. The implementation of a RGBD smart resource is explained in Section 5 by introducing its processing and recognition capabilities. The influence of adaptation in the recognition quality is evaluated in Section 6. Finally in Section 7 some conclusions are summarized and future work is introduced.

## 2. Related Work

The evolution of embedded system capabilities has brought the possibility to perform more complex tasks and to provide smarter services. Therefore, embedded systems can implement self-aware mechanisms as well as routines to adapt their context. Quality measures allow systems to check their performance, detect an undesirable execution context, and warn about system malfunctions.

In order to make the system able to adapt to its/the context, it has to have the proper mechanisms to detect the need/necessity of being adapted. QoS-based communication systems [15], are one of the clearest examples. Through the evaluation of some measures like deadlines or timestamps, among other QoS measures, they offer mechanisms to warn about communication problems such as delays or data loss. As some examples, in [16] a QoS-based application for the enhancement of manufacturing communication networks is introduced, while in [17] the implementation of QoS aware mechanisms for dealing with real-time embedded databases is detailed and the work presented in [18] shows an application of QoS in mobile robotics systems.

Beyond the QoS policies, many other works are designed to achieve a quality measure to evaluate the performance of a certain process. In [19] the concept of Quality of Context (QoC) is introduced as a set of measures which checks the precision, probability of correctness, trustworthiness, resolution, and up-to-dateness of context information. In such way, QoC offers mechanisms for analysing and evaluating the performance according to the current context and allowing one to design quality-aware systems just as detailed in [20]. This kind of qualities is usually oriented to measure the quality that involves only end-point devices.

Once the system has mechanisms to detect an undesirable operation performance, or even a system malfunction, it can execute an adaptation process to solve these issues suited to the current context. The QoS detection mechanisms always lead to the implementation of some adaptation routines. One example is introduced in [21], where a QoS based framework which implements several run time adaptation mechanisms is presented. Another example is also presented in [22] where a QoS adaptation procedure is designed to fit to the different constraints of resource availability and input quality into a decentralized nodes coordinated system. Furthermore, others works have tried to apply machine intelligence tools in combination with QoS to predict failures and force adaptation before the quality decreases [23].

In the case of the QoC, several implementations also provide context adaptation mechanisms. In [24] a detailed research focused on how to adapt services to the current execution context is presented and a middleware to manage this QoC adaptation is proposed. Adaptation is a well-known topic in control systems [25]. Thus, these systems can implement QoC adaptation mechanisms to adapt the control execution to the context in order to enhance the general performance of the system just as is introduced in [26].

To make the adaptation process evolve based on previous decisions, there are many learning algorithms that can be applied [27]. The Support Vector Machine (SVM) tool [28] has been implemented in several works in order to improve the quality of the system by offering adaptive fault diagnosis mechanisms [29,30,31] that allow one to select the most proper system context. One example is presented in [32], where SVM is used to implement a non-linear fuzzy control in order to provide an acceptable control quality.

According to these works, three main key concepts are introduced: the capability to measure the system performance through the quality measures management mechanisms just as QoS or QoC, the need of offering procedures to adapt the system performance to the requirements anytime and, finally, enhance and optimize this adaptation by implementing adaptation algorithms.

## 3. Framework

As presented in the introduction, this contribution is framed into a DCS system in which decentralized devices exchanges information in order to execute control tasks. In this frame, sensors can operate independently or can be established as part of a more complex system, which requires certain knowledge of the environment in order to perform an interaction.

Distributed sensor devices are usually designed to execute data processing and classification mechanisms in order to provide high-level information about their sensing. As a result, both the amount of exchanged data and the bandwidth are reduced by supplying to the DCS only the relevant information.

**Figure 1:**
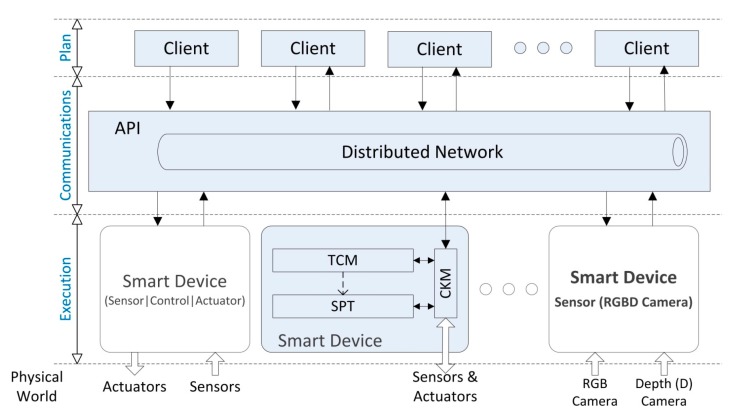
Topology of a distributed control system based on smart devices.

The overview of the framework of our system is detailed in Figure 1. In a down-top approach three different layers are established: execution, communication and plan. The execution layer is carried out by a set of devices with a given computation capabilities which execute different types of tasks: sensorization, processing and actuation. These devices are established as smart devices because they can operate independently and offer high level data management. They are structured in three main components: the Control Kernel Middleware (CKM), the Smart Plugin Topology (SPT) and the Task Configuring Module (TCM) such as will be described in the next section.

The communication layer is provided by an Application Programming Interface (API) based on a Publish/Subscribe distributed network that allows accessing to smart devices transparently. Finally, the client layer is composed by the client processes in order to perform different missions which are achieved through the execution of tasks. Some of these tasks are executed in the distributed smart devices and the communications API offers to clients the mechanisms to configure the parameters of the execution of these tasks and the mechanisms to access and monitor processed data. In this paper, a smart device based on RGBD camera is implemented to test the described DCS.

### 3.1. Smart Devices

**Figure 2:**
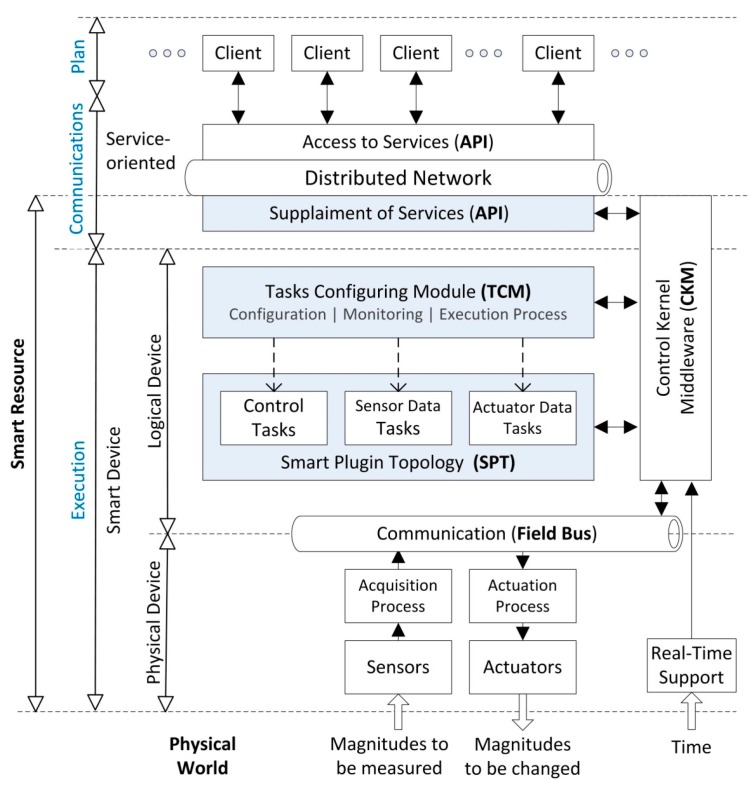
Smart resource: components and relations.

Smart devices (Figure 2) execute their tasks by using a CKM which provides real-time and data management support. The current implementation of the CKM is based in the original proposal described in [33], where is introduced the theoretical background of this control middleware. The CKM also provides field bus communications in order to manage sensors and actuators.

During the acquisition step, the smart device is set to store sensor data at a proper rate which always grants to suit the Nyquist theorem [34]. Raw data is interpreted by a process plugin or a composition of several ones. A plugin is defined as a process function, which extracts information from raw values or the result of another plugin.

The raw data process is based in the work presented in [35] and occurs in three different parts: segmentation, blob detection and feature recognition. First of all the segmentation process allows one to extract same colour and depth regions from the raw image. Next some of these regions are grouped forming image blobs by using the seed region growing (SRG) technique [36]. Finally some shape, size, density and colour characteristics will be analyzed in order to recognize some environment features.

Plugins have been organized within the SPT [37]. The main objective of the SPT is to improve and optimize the processing step defining the plugin configuration and composition capabilities [38] as is detailed in Figure 2. Composition is needed since one plugin output may be useful to another for obtaining more complex information. Therefore, plugins in the SPT can be combined in order to avoid code duplicity and inappropriate use of the system resources. That way, the SPT can dynamically compose a set of plugins to suit the specific requirements of the system.

Different configurations can be specified for parameterizing the process execution. In each case, plugins must be designed to allow some possible configurations enhancing the flexibility of these mechanisms. To select the more suitable plugin configuration for each situation, it must be analyzed the system context by evaluating the Service Requirements (SR), the communication QoS and other quality measures called Quality of Context (QoC) which are relative to the current state of the device and the available resources. QoC will be detailed in next section as end-point metadata quality information. As a result of this evaluation, the execution profile of the smart device is set to perform in the most suitable configuration. The configuration mechanisms are implemented by means of the Task Configuration Module (TCM) that will be fully detailed in next section.

### 3.2. Publish-Subscribe Communications

In the introduction of our framework, the communication has been characterized as a publish/subscribe paradigm. As any implementation of this topology, information is organized by topics. That way each process in the network can publish information in a certain topic in order to send the information between all the processes subscribed to that one. In this way, each device is only aware of the information which concerns its performance. Smart devices are designed to deal with two main types of topics:

- Configuration topics: These topics are used by the client processes to specify the required task of a certain smart device or a group of them.
- Data topics: These topics are used by the clients and smart devices to exchange information.

The quality of communications is an important reliability factor which has to be evaluated in order to ensure proper tasks execution. In this paper QoS policies, such as deadline or lifespan [39], are implemented in order to measure the performance of the communications.

### 3.3. Smart Resources

As stated before, smart devices have been introduced to provide high-level data management, working with well-defined data structures, instead of raw data. In this way, client processes don’t need to use raw values when dealing with sensors and actuators. By adding the communication layer, any process can access this data structures in a homogeneous way through the given API. As a result, a smart device turns into an abstract network resource which offers well defined interaction capabilities for configuring its tasks and requiring or supplying data structures. These resources are named smart resources (Figure 2).

A smart resource realizes its operation in a smart device with service-based distributed communications support. A smart device implements the CKM for supporting the execution of the tasks, the SPT that organizes tasks in plugins and the TCM that configures dynamically the plugins. Communications capabilities are established as a publish/subscribe network as introduced in previous subsections. Following, the description of the TCM will be detailed.

## 4. Task Configuration Module (TCM) in a Smart Resource

How to detect changes in the state of the systems, how to select which scenario suits it more accurately, and how to design the most desirable configuration, are the main contributions of this work. Due to its importance, all these matters will be detailed along this section, where QoS and QoC mechanisms are defined as the most significant tools for achieving the proposal.

### 4.1. Quality Policies: Communication QoS and End-Point QoC

DCS usually implements quality of service (QoS) mechanisms to add reliability and fault tolerance [40] and offer real-time capabilities [41]. Nevertheless, other quality of context (QoC) measures can be evaluated in order to fix the system function. That way, quality of context is defined as some end-point metadata quality policies that will also be analyzed to bring new adaptation mechanisms to the system.

These QoC policies and its meaning could be managed in very different ways depending on the application and the goal. For this reason smart resource services must be developed in order to support different quality policies, in both terms QoS and QoC, bounded by their application needs. That way, during the design of a new control system, smart resource services must be parametrized to suit the requirements of the system. These requirements can be included in one of the following domains:

- Temporal: Related with time values as periods, latency, or delays. Temporal requirements are hard constraints for reliable control system execution.
- Spatial: Lack of memory, memory inconsistency, and data isolation problems could lead to system malfunction.
- Performance Reliability awareness: Awareness of incoherent values, out of bound data, or undesirable combination of system variables, between other, are key evaluators to trigger smart resource reconfiguration to select a more proper scenario.

### 4.2. System Profiles

Control systems can operate in many different execution profiles, ranging from idle mode to the edge of its capacities, executing one or several different tasks. One system, ever with only one well defined task, can face different requirements with different tolerances along the progression of its tasks. That way, each possible situation, with its own requirements, define a new system profile.

More detailed, a certain system profile (SP) is characterized by a particular configuration (*Pg*Mode) of the plugins (*Pg*) defined in the SPT and a set of requirements (*Q*mode) of the Quality (Q)-policies which have to be met.

**Figure 3:**
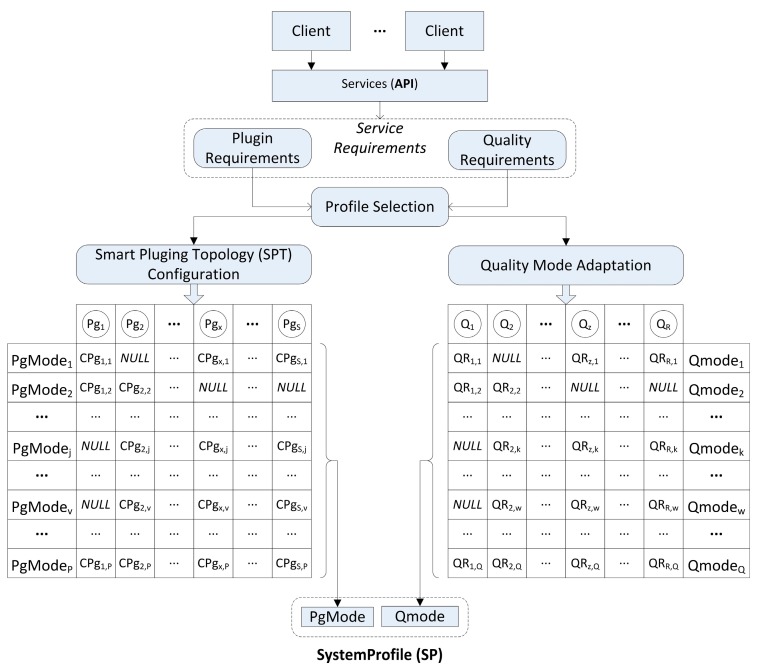
TCM description.

Therefore, the TCM (Figure 3) is composed of a set of possible profiles and its mission is the dynamic selection of the most appropriate profile depending on the service requirements.

As shown in Equation (1), the TCM is formalized as a set of System Profiles SP, which are designed to execute P different process plugins, and to adapt *Q* different quality requirements. That way, a TCM is defined by a set with *P·Q* number of possible system profiles:

(1) $$TCM=\left\{SP_{jk}\left|j=1,\;\ldots ,\;P\right|\;k=1,\;\ldots ,\;Q\right\}$$

Consequently in Equation (2), a system profile SP is defined by a certain Plugin *Pg*Mode, from the given set of P plugins, and a certain quality requirement *Q*mode from the set *Q* requirements. These *Pg*Modes and *Q*modes are respectively defined in the TCM:

(2) $$SP_{jk}=\left\{PgMode_{j},Qmode_{k}\left|j=1,\;\ldots ,\;P\right|\;k=1,\;\ldots ,\;Q\right\}$$

A C*Pg*_xj_ is the particular configuration of the plugin_x_ according to the *Pg*Mode_j_ and *S* is the number of the execution plugins implemented in the SPT:

(3) $$PgMode_{j}=\left\{CPg_{xj}|x=1,\;\ldots ,S\right\}$$

Equation (4) introduces a *Q*mode_k_ as the requirement of the *Q*-policy_z_ defined by *QR_zk_*_,_ where *R* is the number of the *Q*-policies considered in the TCM. That is, *QR_zk_* defines the range of values that are appropriate and acceptable for the *Q*-policy_z_:

(4) $$Qmode_{k}=\left\{QR_{zk}|z=1,\;\ldots ,R\right\}$$

The TCM has to evaluate dynamically if present *Q*-policy values (*Q*’) are meeting the requirements (they are within the ranges specified). In a formal description, any quality policy is evaluated along time t, and is presented as a set of qualities values *Q*Value(t) into the domain above described and referenced in Equation (5):

(5) $$Q\mathrm{Value(t)}=\left\{{Q^{\prime }}_{z}\left(t\right)|z=1,\ldots ,R\right\}$$

### 4.3. Profile Selection

Once the definition of the quality policies, system profiles, and the description of the TCM have been detailed, the process for profile selection will be described. The main purpose of the TCM is to active the most suitable profile of all the possibilities according to the service requirements (plugin requirements and quality requirements). Therefore, active profile will remain while the Configuration of the Plugins (C*Pg*) doesn’t change and Quality Requirements (QR) are fulfilled. If one of these conditions are not satisfied, adaptation mechanisms will change the active profile.

The new active profile is selected by suiting the system execution according to the recent events and the evaluation of each possible profile. This evaluation is calculated by implementing some techniques based on active learning. Therefore, Soft Margins [28] are applied to compute the state of each profile as is introduced in Equation (6):

(6) $$\;EV\_SP_{jk}\left(t\right)=\;w_{jk}\cdot f_{ev}\left(Qmode_{k},\;QValue\left(t\right),\;\xi_{jk}\right)-th\;\left|j=1,\;\ldots ,\;P\right|\;k=1,\;\ldots ,\;Q$$

where $EV\_SP_{jk}\left(t\right)$ is the evaluation of the system profile SP***jk*** at time *t*, and *P*·*Q* is the number of possible profiles. The evaluation function returns a value is between 0 (not suitability) and 1 (full suitability) and it is calculated according to QValue(t) (present quality values), $Qmode_{k}$ (quality requirements defined in the profile jk) and $\xi_{jk}$ (penalization factor for the profile_jk_). The weight value *w_jk_* is a fixed measure for the profile***_jk_*** that allows to modify the result of this evaluation function. Two different profiles with a same result for the *f_ev_* can be differentiated because different weight values. That way, system will lead to the execution of preferred profiles in case of evaluation draw. Finally, *th* is a common threshold value for all profiles which allows to bound the global evaluation for all the profiles in the system.

The value of $\xi_{jk}$ in this equation reflects a penalization factor which avoids the system to oscillate between active profiles. This value is updated, $\xi \prime_{jk}$, by computing the inequation presented in Equation (7) when the expulsion of an active profile takes part due to a policy failure:

(7) $$f_{ev}\left(Qmode_{k},\;QValue\left(t\right),\;\xi_{jk}\right)\geq \left(1-{\xi^{\prime }}_{jk}\right)$$

where $\xi \prime_{jk}$ is the updated value for $\xi_{jk}$ when the profilejk is substituted. According to this, a good evaluation from *f_ev_* will be reflected as a decrement of $\xi \prime_{jk}$ from its previous value $\xi_{jk}$, while a poor evaluation result will reflect an increment of this value. As this evaluation is also conditioned by the penalization value, it prevents the system to oscillate between high and low evaluation results. As will be shown in the results section, it leads to a more stable execution of the system.

The ***f_ev_*** algorithm is detailed using pseudo-code in Algorithm 1, where *QR_zk_* and ${Q^{\prime }}_{z}\left(t\right)$ are the requirement and the present value respectively of the *Q*-policy**_z_** according to the $Qmode_{k}$ of the profile_jk_, and *R* is the number of the Q-policies considered in the TCM.
**Algorithm 1.** Calculating return value of function *f_ev_*1: **function**
***f_ev_*** (Qmode_k_, QValue(t), $\xi_{jk}$).2:  acc **←** 03:  **for** z**←**1 to R **do**4:   **if** Q’_z_(t) = QR_zk_
**then**5:    acc **←** acc + 16:   **end if**7:  **end for**8:  suit **←** acc/n9:  affectedSuit **←** suit * (1 − $\xi_{jk}$).10:  **return** affectedSuit.11: **end function**

**Figure 4:**
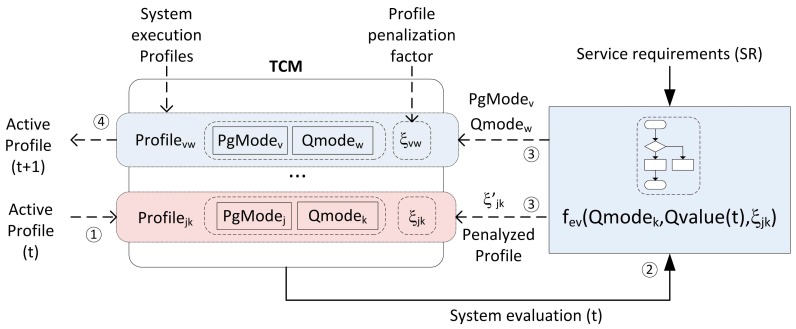
Task configuration selection mechanism implemented based on the service requirements.

The graphical representation of this proposal is shown in Figure 4. In this figure the flow of one step in the profile selection mechanism can be observed. How the quality requirements are conditioning the switch between profiles is also presented. This adaptation mechanism is integrated to suit the profile selection into the smart resource TCM implementation.

## 5. Case of Study: Smart Resource Implementation Based on a RGBD Camera

**Figure 5:**
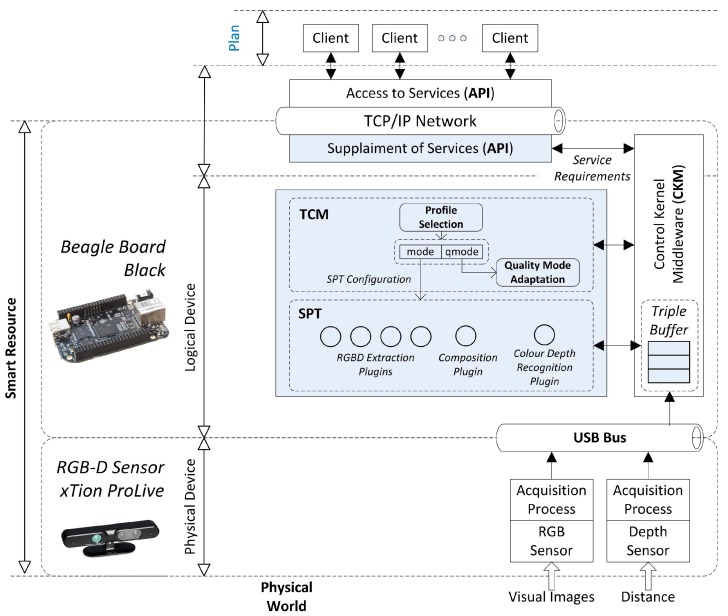
Smart resource implementation based on a RGBD camera.

In this section is introduced a case of study where a smart resource which is designed in order to extract 3D visual information from the environment is implemented. For this purpose, the smart resource will integrate a smart device with an Asus Xtion camera as main component (Figure 5). The Asus Xtion is a RGB-D sensor which is characterized to provide RGB and depth image measurements. A triple buffer [42] implementation ensures it always has fresh data available without interfering with the acquisition.

Two kinds of plugins have been implemented according to the type of the supplied image (RGB or depth). The combination of these will result in a more accurate knowledge of the sensed environment. As a result, the smart resource will produce high-level information structures from the sensor data for being accessible through the communications API by offering distributed services. Therefore, next process plugins are implemented:

- Basic Colour Element Extraction: These plugins extract the information about the elements in the image which are bounded in only one (R, G, B) colour spectre.
- RGB Elements Extraction: As a combination of the previous plugins, all R, G, and B elements are extracted at the same time from the source image.
- Depth Elements Extraction: This plugin uses the depth image to extract object located in a same range of distance from the sensor.
- Colour Depth Elements Extraction: As the most complex plugin, it combines the two previous ones obtaining the colour elements according to their depth values.

**Figure 6:**
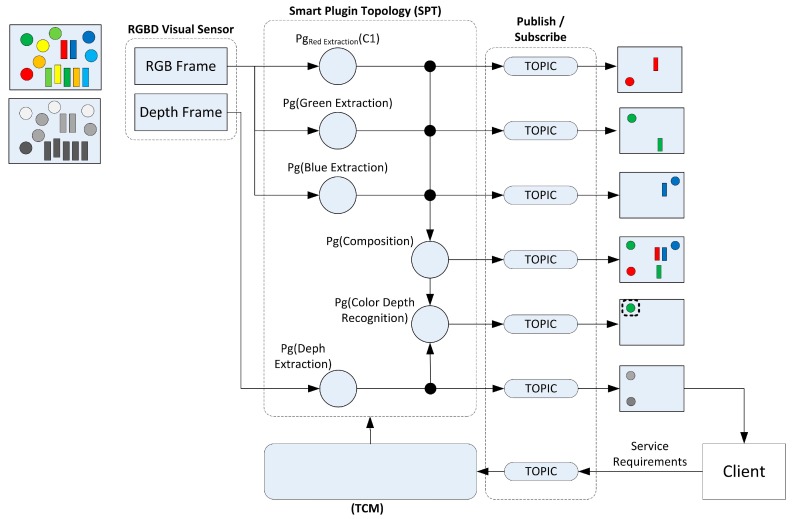
Plugins composition.

The graphical composition of these plugins is detailed in Figure 6. The information obtained as a result of a certain plugin execution is published in a specific topic in order to allow clients to access it.

**Figure 7:**
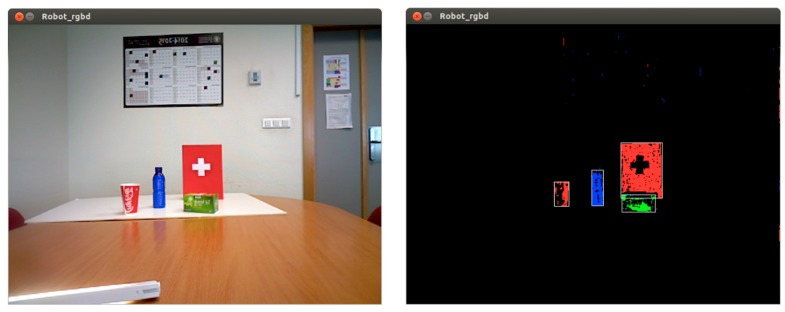
Elements detection.

These plugins can be configured to detect environment elements. Since the scope of this work is not to introduce a recognition system, simple object detection mechanisms have been designed. For this purpose, some basic colour blobs with their depth values are computed, just as is depicted in Figure 7.

The System Plugin Topology (SPT) offers highly parameterizable operation processes through the plugin configuration and composition. Thus, the RGBD camera could be set for working with some different image resolution and different colour formats.

In this implementation quality requirements have been set in terms of both QoS and QoC. As main QoS, the time needed to process the information and publish the result is measured to compare it with a deadline in order to detect unexpected delays on data supply. The evaluated QoC policies are related with the resource usage of the smart device, that way the CPU and memory consumption are measured. To suit the quality requirements each plugin can be configured to perform at different levels of resolution in order to reduce the resources consumption and the response time. Three different levels of resolution are implemented: Video Graphics Array (VGA) (640 × 480), Quarter Video Graphics Array (QVGA) (320 × 240) and Q2VGA (160 × 120).

As has been introduced, a profile is composed by a particular configuration of the plugins of the SPT and some required quality policies. The set of available system execution profiles to be adopted by the current smart resource are defined in Table 1 and Table 2.

**Table 1:** *Pg*Modes for system profiles.

	*Pg*1 = Red Detection	*Pg*_2_ = Green Detection	*Pg*_3_ = Blue Detection	*Pg*_4_ = Depth Detection	*Pg*_5_ = Composition	*Pg*_6_ = Colour Depth Recognition
*Pg*Mode_j_	{null, VGA, QVGA, Q2VGA}	{null, VGA, QVGA, Q2VGA }	{null, VGA, QVGA, Q2VGA }	{null, VGA, QVGA, Q2VGA}	{null, *Pg*_1_ + *Pg*_2_ + *Pg*_3_}	{null, *Pg*_4_ + *Pg*_5_ }

**Table 2:** Possible *Q*modes for system profiles.

	*Q*_1_ = CPU [min.,max.]	*Q*_2_ = Memory	*Q*_3_ = Deadline
*Q*mode_1_	[20%, 40%]	15 MB	50 ms
*Q*mode_2_	[40%, 60%]	15 MB	50 ms
*Q*mode_3_	[60%, 80%]	15 MB	50 ms

## 6. Experiments and Results

**Figure 8:**
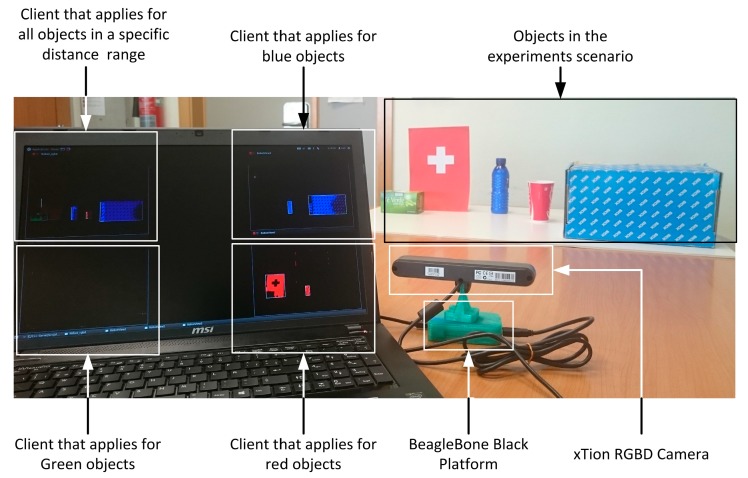
RGBD smart resource based on XTion and BeagleBoard working.

A set of tests were designed in order to validate the implemented RGBD smart resource (Figure 8). In these tests, four different clients publish on the configuration topic in order to apply for the execution of a new type of plugin. Each client applies for a specific plugin in every execution and the number of plugins and their configurations will change during the tests. Along these executions, qualities will be monitored, in addition with the active system profile in each time and the penalization factor for each one of them.

**Figure 9:**
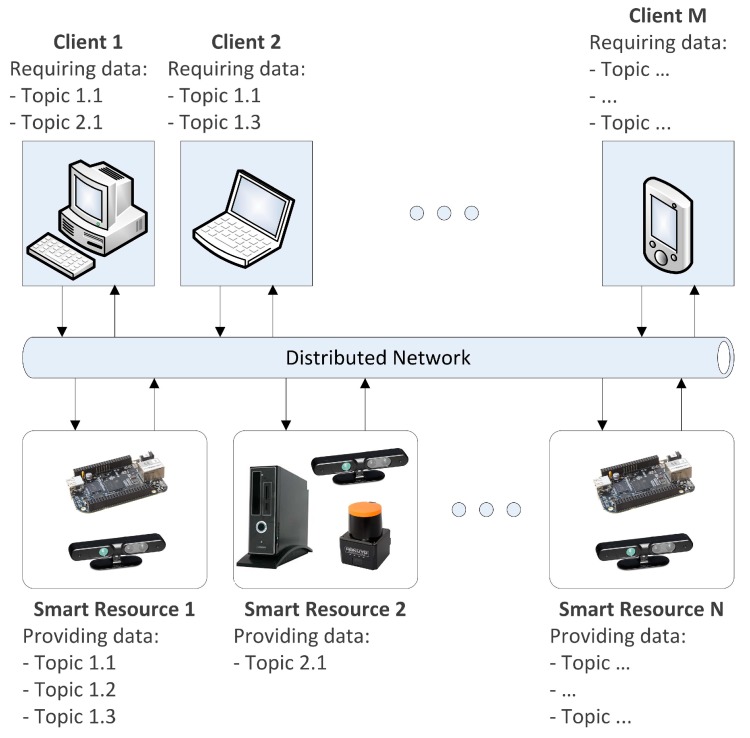
Scalability of the system.

Figure 9 shows the scalability of the system. Both, clients and smart resources may be added to the distributed system by using the publish/subscribe infrastructure based on topics. In the case of the experiments presented in this paper, the client scalability is tested in order to validate the configuration selection mechanism proposed for the smart resource.

**Figure 10:**
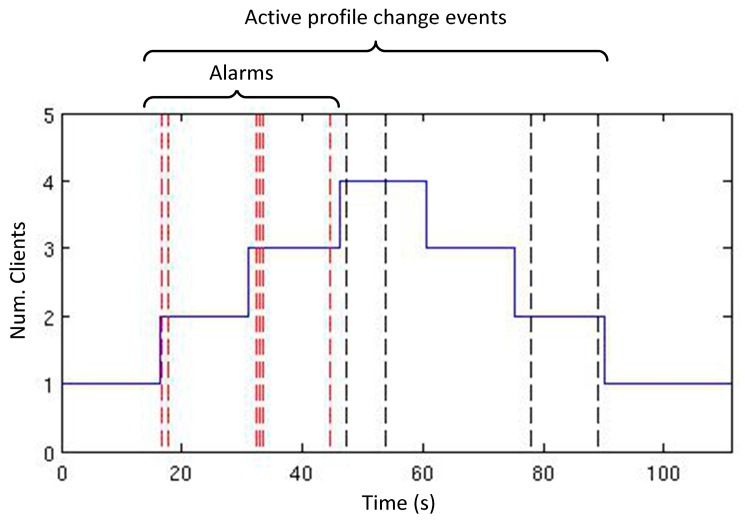
Number of clients along experiments and alarms produced.

**Figure 11:**
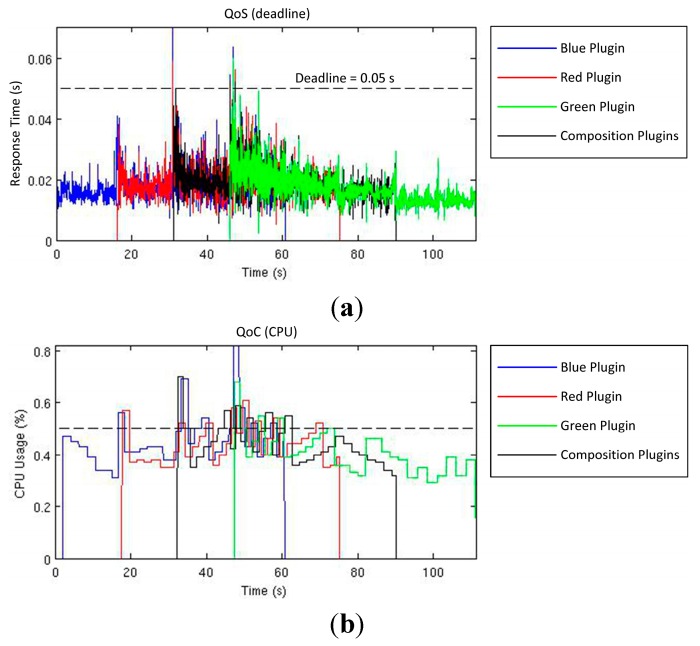

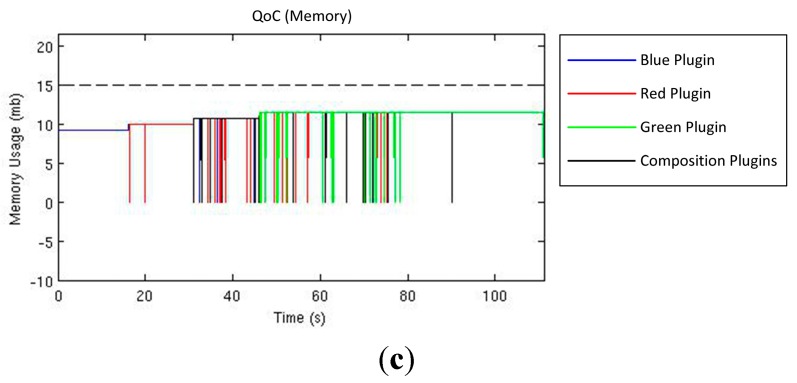
QoS and QoC values along tests performed: deadline (**a**); CPU load (**b**); and memory usage (**c**).

**Figure 12:**
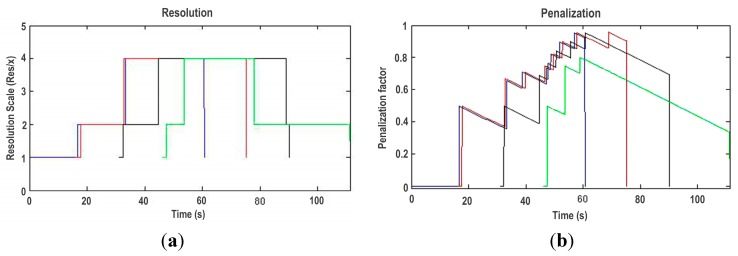
Evolution of the plugin image resolution (**a**); and the penalization factor (**b**).

**Figure 13:**
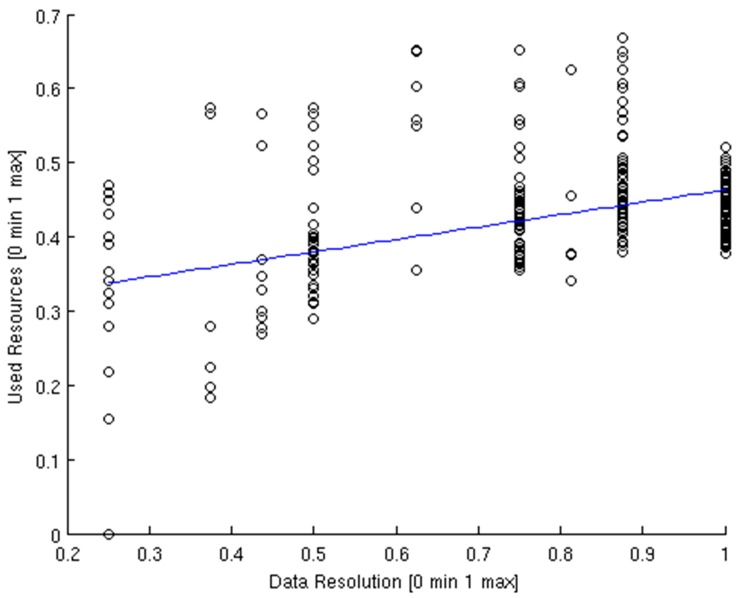
Data resolution *vs.* used resources.

**Figure 14:**
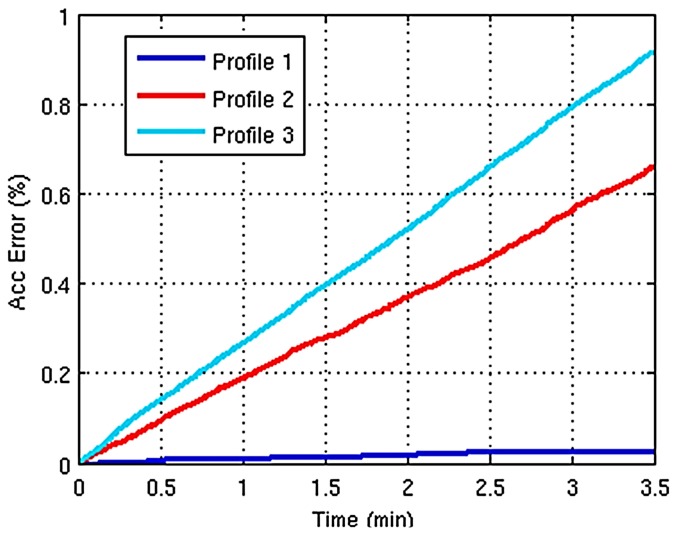
Accumulated errors.

In the experiment, testbeds consist on one to four clients that are executed sequentially 180 times. The first client requests for blue objects, the second one requests for red objects, the third client requests for objects placed in a specific distance range and, finally, the last one requests for green objects. After that, in the same order, clients request the end of each service requested. As a consequence of each client request, necessary plugins to serve the request are started in one RGBD smart resource. The execution of new plugins can produce changes in the quality values and can trigger an alarm if these values exceed required quality ranges. As a result, the TCM adapts dynamically the configuration of the plugins by changing the active system profile due to the number of active clients (Figure 10). The evolution of this execution test is detailed in Figure 11 and Figure 12. And the consequences of the execution in the QoC (memory and CPU load) are detailed in Figure 13 and Figure 14. Vertical lines in Figure 10 shows in the horizontal axis the moments in which relevant events happen. Horizontal axis of the Figure 10 corresponds with the horizontal axis of Figure 11, Figure 12, Figure 13 and Figure 14.

In Figure 11 the evaluated qualities and the established limits for each value are shown. As can be observed, the deadline (Figure 11a) and the CPU (Figure 11b) measurements are the most critical qualities, due to existence of outline values beyond the specified bound in each case. The memory usage is permanently below the limit defined (Figure 11c). Memory is depicted as the full usage accumulated by every plugin in the processor. Whenever a plugin changes its configuration (for example, as a consequence of an alarm) it leaves the processor and reduces its memory load to zero, for this reason every vertical line in Figure 11c represents a plugin’ context switch.

When quality limits are exceeded, the CKM detects the event and the corresponding alarm is triggered. As a result, the TCM evaluates the profiles and selects a new active profile decreasing the resolution of the images (Figure 12a). Table 3 shows the evolution of these changes: active plugins and *Pg*Modes associated to the different active profiles with their corresponding image resolution.

**Table 3:** Evolution of *Pg*Modes

	Client 1	Client 2	Client 3	Client 3	Client 3	Client 4
*Pg*Modes (Trigger Driven)	*Pg*_3_ = Blue Detection	*Pg*_1_ = Red Detection	*Pg*_4_ = Depth Detection	*Pg*_5_ = Composition	*Pg*_6_ = Colour Depth Recognition	*Pg*_2_ = Green Detection
***Pg* Mode_1_**	VGA					
***Pg* Mode_2_**	VGA	VGA				
***Pg* Mode_3_**	QVGA	VGA				
***Pg* Mode_4_**	QVGA	QVGA				
***Pg* Mode_5_**	QVGA	QVGA	VGA	*Pg*_1_ + *Pg*_2_ + *Pg*_3_	*Pg*_4_ + *Pg*_5_	
***Pg* Mode_6_**	QVGA	QVGA	QVGA	*Pg*_1_ + *Pg*_2_ + *Pg*_3_	*Pg*_4_ + *Pg*_5_	
***Pg* Mode_7_**	Q2VGA	Q2VGA	QVGA	*Pg*_1_ + *Pg*_2_ + *Pg*_3_	*Pg*_4_+ *Pg*_5_	
***Pg* Mode_8_**	Q2VGA	Q2VGA	QVGA	*Pg*_1_ + *Pg*_2_ + *Pg*_3_	*Pg*_4_ + *Pg*_5_	VGA
***Pg* Mode_9_**	Q2VGA	Q2VGA	Q2VGA	*Pg*_1_ + *Pg*_2_ + *Pg*_3_	*Pg*_4_+ *Pg*_5_	QVGA
***Pg* Mode_10_**	Q2VGA	Q2VGA	Q2VGA	*Pg*_1_ + *Pg*_2_ + *Pg*_3_	*Pg*_4_ + *Pg*_5_	Q2VGA
***Pg* Mode_11_**		Q2VGA	Q2VGA	*Pg*_1_ + *Pg*_2_ + *Pg*_3_	*Pg*_4_+ *Pg*_5_	Q2VGA
***Pg* Mode_12_**			Q2VGA	*Pg*_1_ + *Pg*_2_ + *Pg*_3_	*Pg*_4_ + *Pg*_5_	Q2VGA
***Pg* Mode_13_**			QVGA	*Pg*_1_ + *Pg*_2_ + *Pg*_3_	*Pg*_4_ + *Pg*_5_	Q2VGA
***Pg* Mode_14_**			QVGA	*Pg*_1_ + *Pg*_2_ + *Pg*_3_	*Pg*_4_ + *Pg*_5_	QVGA
***Pg* Mode_15_**						QVGA
***Pg* Mode_16_**						VGA

The changes between image resolutions according to the requirements of the system are detailed in Figure 12a. As can be shown, a certain number of plugins leads to a stable configuration of plugins, but at the same time the TCM is always evaluating the resources in order to increase again the resolution. This resource evaluation is conditioned by the evolution of the penalization factor as depicted in the respective graph (Figure 12b). In Section 4.3 the function of this penalization value has been theoretically introduced, which in this test proves to suit the dynamics of the system, leading to a resolution change only when the context meets the requirements.

The results of this test, depicted in Figure 10, Figure 11 and Figure 12, are numerically analyzed in Table 4 where a quantitative analysis of the evolution of the system variables according to the number of active clients, which reflects in fact the number of active plugins, is gathered. As the numbers of clients are augmented, the activation time of higher resolutions decreases whereas the deadline, CPU and memory measures are augmented. Once the system resources are employed to the edge of its capabilities, the number of alarms produced by the system is increased. As the number of alarms increases, the global penalization of the system augments. A significant increment on the alarms, and consequently the penalization, can be interpreted as an approach to the maximum system resource usage according to the specified quality bounds.

**Table 4:** Quantitative study of the system evolution.

N. Clients	Resolution	% Active	Events	Alarms	Deadline	CPU	Memory	Penalization
	VGA	0.4637						
1	QVGA	0.5631	0	0	0.0142	34.34	1.048	0.24
	Q2VGA	0.0000						
	VGA	0.0321						
2	QVGA	0.6877	4	0	0.0169	39.11	1.069	0.54
	Q2VGA	0.2800						
	VGA	0.0160						
3	QVGA	0.1860	4	0	0.0190	43.97	1.089	0.69
	Q2VGA	0.7980						
	VGA	0.0220						
4	QVGA	0.1090	2	13	0.0221	48.27	1.130	0.76
	Q2VGA	0.8660						

In order to analyse the global performance of the system a scatterplot in which the average resolution of the 180 experiments performed during this test is compared with its correspondent usage of system resources is depicted in Figure 13. Although the point dispersion is significant, it can be set a lower limit which can be interpreted as the minimum usage of resources that can be obtained with a specific data resolution. According to the point dispersion, the deterministic behaviour of the system must be studied. In order to test the repeatability of its execution, a specific test will be repeated for a previous analysis of its statistical characteristics.

In Table 5 are analyzed the variance and the standard deviation of the global resolutions and qualities measured throw the 180 experiments. As the values obtained are very similar, it can be said that the experiment is repeatable and, consequently, validate the results scientifically.

**Table 5:** Variance and standard deviation.

	Active Resolution	QoS (Deadline)	QoC (CPU & Memory)
**Variance**	0.0078	0.00000025	0.007799
**Standard Deviation**	0.0709	0.00047322	0.007280

The presented adaptation mechanism has been characterized as a useful tool to align the system performance to the system context. However, a decrease on the process quality in order to fit the dynamic requirements could lead to several failures on the development of their tasks. In this case of study, a down-scale in the image resolution can avoid the smart resource to perceive or recognize an environment object. For this reason, a set of experiments in which is characterized the influence of this adaptation in the quality of the information has been designed.

During each experiment, the smart resource will be forced to work with a fixed profile in order to determine the quality of the recognition in each one. Every profile is configured to use a different resolution: VGA in Profile 1, QVGA in Profile 2, and Q2VGA in Profile 3. During each experiment when the perception fails (an environment element is not detected) and the recognition fails (a detected element is not recognized) will be annotated in order to summarize the obtained error in each profile, Table 6 shows the total of perception and recognition fails in each profile. This table also adds information about the false positives, defined in these cases as an environment element that has been erroneously recognized as an object. Finally, Figure 14 shows the evolution of the accumulated error along the experiment for each profile. As expected, Profiles 2 and 3 offers much lower reliability than the Profile 1 but the total error is not significant.

**Table 6:** Table of accumulated errors.

Scenarios	Profile 1	Profile 2	Profile 3
**Perception Fail %**	0.0060	0.0230	0.2740
**Recognition Fail %**	0.0000	0.1752	0.013
**False Positive %**	0.0020	0.0000	0.0000
**Total Error %**	0.0027	0.0661	0.0957

## 7. Conclusions

In this paper a smart resource which allows to apply for high-level information through a set of distributed services has been introduced. This allows devices to perform more complex tasks (like sensor or actuator data adequacy) through the SPT. In the case of a RGBD sensor, it allows us to apply it for detected objects without requiring any knowledge of computer vision mechanisms.

Services are configured to fit some quality policies in order to not to exceed the resource usage limit of each service in the smart device (the cyber-physical device, not the smart resource abstraction).

TCM has been tested in order to analyse the dynamic adaptation of the SPT in order to fit the different services required along time through the management of Q-alarms.

The system is limited by its available resources. A strict demand of quality could lead to a constant failure to meet the quality policies. Nevertheless the system will be adapted to offer the more adequate execution profile between all the available profiles. In some cases this quality adaptation reflects a decrease of the data quality in order to fit the quality policies.

As future work, it would be convenient to continue the study about the effect of this adaptation in the quality of the provided data by using a partitioned smart resource with mixed criticality services. Additionally, more experiments with more than one smart resource distributing to several clients can demonstrate the influence of network features, like bottlenecks or throughput penalisation, in a distributed system based on smart resources.
